# Low IgM Levels in Adult IEI: Classification Challenges and Clinical Implications

**DOI:** 10.1007/s10875-026-02017-x

**Published:** 2026-04-14

**Authors:** Reyhan Gumusburun, Kasım Okan, Ersin Akdaglı, Onurcan Yıldırım, Hatice Serpil Akten, Sinem Inan, Gulhan Demiroglu, Zuleyha Galata, Ragıp Fatih Kural, Hasibe Aytac, Yusuf Ozeki, Eda Aslan, Umitcan Ates, Kutay Kırdok, Meryem Irem Toksoy Senturk, Nalan Gulsen Unal, Derya Demir, Nur Soyer, Mehmet Soylu, Funda Elmas Uysal, Ayca Aykut, Asude Durmaz, Ceyda Tunakan Dalgıc, Aytul Zerrin Sin, Omur Ardeniz

**Affiliations:** 1 Department of Internal Medicine, Division of Allergy and Clinical Immunology, Ege University Medical Faculty, Izmir, Türkiye Turkey; 2 Department of Internal Medicine, Ege University Faculty of Medicine, Izmir, Turkey; 3 Faculty of Medicine, Department of Internal Medicine, Division of Gastroenterology, Ege University, Izmir, Turkey; 4 Department of Pathology, Ege University Faculty of Medicine, Izmir, Turkey; 5 Faculty of Medicine, Department of Internal Medicine, Division of Hematology, Ege University, Izmir, Turkey; 6 Faculty of Medicine, Department of Microbiology, Ege University, Izmir, Turkey; 7 Faculty of Medicine, Department of Pulmonary Diseases, Ege University, Izmir, Turkey; 8 Faculty of Medicine, Department of Medical Genetics, Ege University, Izmir, Turkey

**Keywords:** Inborn errors of immunity, Low serum IgM, Selective IgM deficiency, Adult immunodeficiency, Clinical heterogeneity, Immunological phenotypes

## Abstract

**Purpose:**

Reduced serum IgM is increasingly encountered in adults evaluated for inborn errors of immunity (IEI), yet its clinical relevance and diagnostic utility remain uncertain. While some patients meet criteria for selective IgM deficiency (SIgMD), others remain unclassified, reflecting significant diagnostic heterogeneity. This study aimed to characterize adult IEI patients with low serum IgM and to assess the prognostic value of IgM levels for clinical outcomes.

**Methods:**

Among 378 adult IEI patients followed at a tertiary immunology center, 43 individuals (11.4%) with low serum IgM were included. Clinical, immunological, genetic, and treatment data were analyzed, and associations between IgM levels and clinical outcomes were assessed using regression and ROC analyses.

**Results:**

The median age was 58 years, and most patients were referred following incidental detection of hypogammaglobulinemia. Recurrent infections and allergic diseases were the most common manifestations and generally preceded IEI diagnosis. While most clinical features occurred before diagnosis, hepatobiliary disease, lymphoproliferation, osteoporosis, and malignancies showed variable timing. Median IgM memory B-cell and naïve CD8⁺ T-cell percentages were below the reference range, with reductions observed in 52.9% and 53.8% of patients, respectively, and elevated IgE levels in 35.7%. Pathogenic or likely pathogenic variants were identified in a subset of patients, often without clear genotype–phenotype correlation. Serum IgM levels demonstrated limited predictive value for clinical outcomes.

**Conclusion:**

Low serum IgM in adults reflects a heterogeneous immunodysregulatory state rather than a uniform immunodeficiency. Serum IgM levels alone provide limited discriminatory and prognostic value, underscoring the need for integrated clinical and immunological assessment in diagnosis and management.

**Supplementary Information:**

The online version contains supplementary material available at 10.1007/s10875-026-02017-x.

## Introduction

Immunoglobulin M (IgM) is the earliest antibody isotype expressed during B-cell development and plays a key role in early humoral immune responses. Membrane-bound IgM functions as the antigen receptor on naïve B cells, whereas secreted IgM circulates predominantly as a pentameric immunoglobulin complex [1–3]. Owing to its multimeric structure, IgM exhibits high antigen avidity and is the most efficient immunoglobulin isotype in activating the classical complement pathway and mediating rapid pathogen neutralization.

Beyond antimicrobial defense, IgM recognizes a broad range of targets, including altered self-antigens, apoptotic debris, and non-protein motifs. Through these properties, natural IgM contributes to immune homeostasis, apoptotic cell clearance, and regulation of inflammatory responses, with accumulating evidence supporting its role in maintaining immune tolerance and preventing autoimmune pathology [2–6].

Serum IgM measurement is clinically relevant in the evaluation of immune deficiencies, autoimmune disorders, infections, and hematologic malignancies. In adults, normal serum IgM concentrations are generally reported to range between approximately 50 and 200 mg/dL, although reference intervals vary across laboratories and populations [7, 8]. This variability complicates the interpretation of isolated IgM abnormalities and represents a major source of diagnostic uncertainty in adult patients.

Selective IgM deficiency (SIgMD) was first described in case reports nearly 60 years ago and was formally incorporated into the International Union of Immunological Societies (IUIS) classification of inborn errors of immunity (IEI) in 2017 [9, 10]. The prevalence of complete SIgMD has been reported to range between 0.03 and 0.37% in the general population, whereas rates as high as 0.07–2.1% have been observed in immunodeficiency clinics [11]. According to the criteria of the European Society for Immunodeficiencies (ESID), SIgMD is defined by a marked reduction or absence of serum IgM in the presence of normal IgG, IgG subclasses, and IgA levels, as well as preserved IgG vaccine responses; secondary causes, T-cell defects, and other defined immunodeficiencies must be excluded [4]. However, these exclusion-based criteria may fail to capture a subset of affected individuals, leading to underdiagnosis [12]. Reflecting these limitations, the 2024 update of the IUIS classification, which now encompasses 559 inborn errors of immunity, proposed a simplified definition of SIgMD based primarily on absent serum IgM levels and its classical association with susceptibility to bacterial infections [13, 14]. Nevertheless, reliance on serum IgM concentration alone remains challenging due to age-dependent variation, inter-laboratory differences, and the absence of standardized diagnostic thresholds [15]. These evolving definitions underscore the lack of consensus regarding diagnostic thresholds and raise concerns that clinically relevant cases may remain unrecognized.

Selective IgM deficiency is a rare and often underrecognized immunodeficiency characterized by heterogeneous clinical manifestations. The clinical spectrum ranges from asymptomatic individuals to patients presenting with recurrent infections (reported in up to ~ 80% of cases), allergic manifestations (~ 40%), autoimmune diseases (~ 10–15%), and, less frequently, malignancy [11, 16–18]. Despite increasing recognition of this condition, the immunological correlates, genetic background, and clinical implications of isolated low IgM in adults remain insufficiently characterized.

In this context, the present study aimed to comprehensively characterize the clinical, immunological, and genetic features of adults presenting with reduced serum IgM levels. By stratifying patients according to IgM concentrations, we sought to explore associations with clinical phenotypes and laboratory parameters, evaluate the discriminatory value of IgM thresholds, and provide insights relevant to diagnostic refinement and risk stratification.

## Methods

### Participants / Grouping

The study population comprised 378 adult patients with IEI followed at the Immunology and Allergy Center of Ege University Faculty of Medicine between January 1, 2015 and December 30, 2024. Patients with T-cell defects, or classified primary antibody deficiencies were excluded [19]. Secondary causes of hypogammaglobulinemia were systematically screened for and excluded during patient selection through a detailed review of clinical records. Potential secondary factors, including medication exposure, chronic infections, nutritional deficiencies, protein loss, extremes of age, and hematologic malignancies, were assessed. Serum total protein and albumin levels were measured in all patients as part of routine laboratory evaluation. Urinalysis was performed in all cases, and no patient demonstrated proteinuria suggestive of nephrotic syndrome. Patients presenting with gastrointestinal symptoms underwent endoscopic evaluation as part of routine clinical care. None demonstrated hypoalbuminemia or endoscopic findings suggestive of active protein-losing enteropathy. Patient classification (selective IgM deficiency and unclassified primary antibody deficiency (unPAD)) was performed according to the 2019 ESID registry working definitions [20].

Patients with persistently reduced serum IgM levels < 50 mg/dL constituted the final study cohort (*n* = 43). Persistently reduced IgM was defined as serum IgM < 50 mg/dL confirmed on at least two separate measurements during follow-up. As this was a retrospective study, IgM measurements and other clinical and laboratory variables were extracted from existing medical records and reflected routine clinical follow-up. Measurements were not obtained at predefined standardized intervals. Baseline laboratory parameters were defined as measurements obtained at the time of first evaluation at our center. Patients were routinely followed every three months during the first year. Thereafter, follow-up visits were scheduled at intervals ranging from three months to one year, depending on clinical status and disease course.

Collected variables included demographic characteristics (age, sex, follow-up status, family history, consanguinity and reason for referral), clinical presentation, age at IEI diagnosis, and treatment data (immunoglobulin replacement therapy, antimicrobial prophylaxis, immunosuppressive use). Immunological evaluation comprised serial IgM measurements, IgG and subclasses, IgA, IgE, routine laboratory tests, autoantibodies, lymphocyte subsets, advanced B- and T-cell phenotyping, isohemagglutinins, and vaccine responses. Microbiological and allergic evaluation data were also recorded. All data were entered into a structured database for analysis.

Immunoglobulin replacement therapy (IgRT) was initiated according to institutional clinical practice, particularly in patients with frequent or severe infections who showed an inadequate response to antimicrobial prophylaxis. IgRT was also considered in patients with impaired protective antibody responses despite vaccination (e.g., pneumococcal vaccination) or in the presence of accompanying immunoglobulin abnormalities such as IgG subclass deficiency or IgA deficiency. Clinical burden was assessed using annual patient-days, defined as the cumulative number of days contributed by patients during the observation period within a given year. This metric was calculated by summing the total follow-up days for all patients and normalizing the value per year to account for differences in individual follow-up duration. Follow-up duration was calculated from the first evaluation to the last documented clinical contact. Clinical manifestations were categorized according to their timing relative to IEI diagnosis.

Patients were stratified according to the highest recorded serum IgM level during follow-up to reduce the impact of transient fluctuations. Both the lowest and highest IgM values recorded during follow-up were analyzed. Given the absence of standardized IgM cut-offs, three complementary stratification strategies were applied: (i) analyzer-based cut-offs (≤ 25 mg/dL vs. >25–<50 mg/dL), (ii) literature-based thresholds (< 30 mg/dL vs. ≥30–<50 mg/dL), and (iii) institutional reference range–based cut-offs (< 37 mg/dL vs. ≥37–<50 mg/dL) [16, 21 ].This approach enabled systematic evaluation of clinically relevant IgM thresholds in relation to clinical and immunological outcomes.

### Laboratory Analyses

Venous blood samples were processed within two hours of collection. Serum was separated by centrifugation at 4000 g for 10 min. Routine biochemical parameters were measured photometrically using the Cobas 8000-C702 analyzer (Roche Diagnostics, Switzerland). Complete blood counts were performed within one hour on EDTA-anticoagulated samples using an automated hematology analyzer (Sysmex XN series, Kobe, Japan). Serum immunoglobulin levels (IgG, IgA, IgM) were quantified by immunoturbidimetry on the same platform and interpreted according to age-adjusted reference ranges (normal IgM: 37–286 mg/dL). IgM values below the analytical detection limit were recorded using the reported threshold (< 25 mg/dL or < 17 mg/dL, depending on the analyzer). Both lowest and highest IgM values during follow-up were analyzed.

### Flow Cytometric Immunophenotyping

Peripheral blood mononuclear cells were isolated by Ficoll density gradient centrifugation. Surface and intracellular staining was performed using fluorochrome-conjugated monoclonal antibodies following standard protocols. Data were acquired on a Beckman Coulter Navios EX flow cytometer and analyzed using Kaluza software. Major lymphocyte populations (T, B, NK, NKT cells) and detailed B- and T-cell subsets were evaluated, including naïve, switched memory, IgM memory B cells, transitional B cells, plasmablasts, CD21^low B cells, and naïve and memory CD4⁺ and CD8⁺ T-cell subsets. Reference ranges were based on published population data [22–26].

### Specific Antibody Responses

Anti-tetanus toxoid IgG levels were measured by ELISA (Euroimmun until May 2024; IDS thereafter). Protective immunity was defined as > 0.5 IU/mL (Euroimmun) or ≥ 0.1 IU/mL (IDS). Patients with insufficient baseline immunity received booster vaccination, and a ≥ 4-fold increase or achievement of protective levels was considered an adequate response.

Serum IgG antibodies against Streptococcus pneumoniae capsular polysaccharides were measured by ELISA (ELIZEN Pneumococcus IgG until 2023; BioVendor TestLine thereafter). Pneumococcal immunization was performed using the 23-valent pneumococcal polysaccharide vaccine (PPSV23). Non-protective levels were defined as < 250 mU/mL or < 7 U/mL, respectively. Functional responses were assessed in paired pre- and post-immunization sera obtained 4–6 weeks apart, with an adequate response defined as attainment of protective thresholds or a ≥ 4-fold increase from baseline.

### Isohemagglutinins and Allergy Assessment

Anti-A and/or anti-B titers were determined from serum or plasma obtained from EDTA-anticoagulated blood after ABO group confirmation. Serial twofold dilutions were tested with A1 or B reagent red cells using the Ortho BioVue^®^ column agglutination system, which primarily detects naturally occurring IgM isohemagglutinins. After 30-minute incubation at room temperature, the highest dilution showing agglutination was recorded as the titer. Based on established reference standards, titers ≥ 1:32 were considered significant; in group O patients, both anti-A and anti-B titers were required to be ≥ 1:32 to indicate an adequate response.

Allergy and Atopy Assessment: Skin prick testing was performed using standardized aeroallergen extracts (pollens, house dust mites, moulds, animal dander, and natural rubber latex) with physiological saline and histamine as negative and positive controls. A wheal ≥ 3 mm larger than the negative control after 20 min was considered positive. Allergen-specific IgE to nine aeroallergens was measured using the ImmunoCAP assay, with values ≥ 0.35 kU/L regarded as positive and classified according to manufacturer-recommended classes.

### Genetic Evaluation

Genetic testing was performed in selected patients based on clinical indication. Genomic DNA was extracted from peripheral blood samples using the MagNA Pure 96 DNA and Viral NA Small Volume Kit (Roche Diagnostics) according to the manufacturer’s instructions. Depending on the clinical context, targeted gene panel sequencing, clinical exome sequencing (CES), or whole exome sequencing (WES) was performed. Target enrichment was carried out using the TWIST custom gene panel, TWIST Clinical Exome Solution Panel v2, or the KAPA HyperExome Kit (Roche™), respectively. Sequencing was performed on the MGI sequencing platform (DNBSEQ-G400) following standard library preparation and sequencing protocols. Bioinformatic analysis included alignment to the human reference genome and variant calling using validated analysis pipelines. Detected variants included single nucleotide variants (SNVs) and copy number variations (CNVs). Variant interpretation was performed according to the standards and guidelines of the American College of Medical Genetics and Genomics and the Association for Molecular Pathology (ACMG-AMP) [27, 28]. Variants were classified as pathogenic, likely pathogenic, variant of uncertain significance (VUS), likely benign, or benign based on available evidence including population frequency databases, computational prediction tools, published literature, and reported disease associations. Because sequencing was performed using DNA derived from peripheral blood, the possibility of somatically acquired variants related to age-associated clonal hematopoiesis was considered during variant interpretation, particularly for variants detected in genes known to be associated with clonal hematopoiesis.

### Statistical Analysis

Categorical variables were expressed as frequencies and percentages, and continuous variables as mean ± SD or median (min–max), as appropriate. Group comparisons were performed using Chi-square or Fisher’s exact test for categorical variables and Mann–Whitney U or Kruskal–Wallis tests for continuous variables. ROC curve analysis with Youden index optimization was used to evaluate IgM cut-off performance. Correlations were assessed using Spearman’s rank coefficient, and logistic regression was applied to evaluate independent associations. Statistical significance was set at *p* < 0.05. Analyses were conducted using R software (version 4.0.5).

## Results

Among 378 adult IEI patients followed at our center, 43 (11.4%) met the inclusion criteria and were included in the final analysis. The median age was 58 years (range: 21–79), 51.2% were female, and the median age at IEI diagnosis was 55 years. At last follow-up, 97.7% of patients were alive, with ongoing follow-up in 58.1%. The median follow-up duration was 10 months (IQR: 6–22; range: 3–96). Parental consanguinity and a family history of IEI were present in 23.3% and 14% of patients, respectively. Low immunoglobulin levels were the most common reason for referral, whereas recurrent infections were the most frequent presenting symptom. Positive isohemagglutinin titers were observed in 23.3% of patients, with positive pneumococcal and tetanus vaccine responses in 46.5% and 69.8%, respectively. Among patients who underwent vaccination, pneumococcal responses were evaluated following PPSV23 immunization, with 20 of 22 patients (90.9%) demonstrating an adequate response, while tetanus vaccine responses were adequate in 30 of 36 evaluable patients (83.3%). Patients had a median of four comorbidities (IQR: 3–6), and 41.9% had received immunosuppressive therapy (Table 1).

**Table 1:** Demographic, clinical, immunological, and autoantibody characteristics of the study population (*n* = 43)

Characteristic	Median (Min–Max); Mean ± SD and/or *n* (%)	Characteristic		*n* (%)
Age (years)	58 (21–79); 56.4 ± 14.4	Gender	Female	22 (51.2)
			Male	21 (48.8)
Age at IEI Dx (years)	55 (21–79); 52.5 ± 16.1	Life status	Alive	42 (97.7)
			Deceased	1 (2.3)
Annual Patient-Days	0 (0–32); 2.4 ± 6.4	Follow-up Status	Present	25 (58.1)
			Absent	18 (41.9)
Age at Prophylactic Antibiotic Initiation (years)	49 (25–66); 46.3 ± 12.0; 12(27.9)	Parental consanguinity	Present	10 (23.3)
			Absent	28 (65.1)
Age at IGRT Initiation (years)	56 (27–72); 51.3 ± 16.2; 9 (20.9)	Family History of IEI	Present	6 (14)
			Absent	32 (74.4)
Comorbidity Count in Patients	4 (1–11); 4.4 ± 2.3	IgRT exposure	None	34 (79.1)
			Prior	7 (16.3)
			Ongoing	3 (6.97)
Blood group				
A / B	18 (41.9)/ 5 (11.6)	Type of referral	Consultation	28 (65.1)
AB/ O	2 (4.7)/ 11 (25.6)		Transfer*	4 (9.3)
Not performed	7 (16.3)		Self-referral	11 (25.6)
Isohemagglutinin titration		Referral indication	Low immunoglobulin levels	30 (69.8)
Positive (≥ 1:32)	10 (23.3)		Recurrent infections	9 (20.9)
Low-positive (< 1:32)	19 (44.2)		Skin involvement	3 (7)
Not performed	14 (32.6)		Positive family history	1 (2.3)
Isohemagglutinins		Initial Symptom	Recurrent infections	21 (48.8)
Anti-A titer in group B	16 (4–32); 18.4 ± 13.1		Skin manifestations	10 (23.3)
Anti-B titer in group A	16 (2–512); 52.8 ± 128.6		Diagnosis via screening	6 (12)
Anti-A titer in group O	32 (16–256); 67.6 ± 79.9		Musculoskeletal complaints	4 (9.3)
Anti-B titer in group O	32 (0–128); 48.0 ± 48.7		Allergic nasal symptoms	2 (4.7)
Pneumococcal vaccine response		Immunosuppressive Agent	Corticosteroids (Methylprednisolone, Prednisolone)	18 (41.9)
Positive	20 (46.5)		Antimetabolites (Azathioprine, Methotrexate, Mycophenolate mofetil)	6 (14)
Negative	2 (4.7)		Alkylating agents (Cyclophosphamide)	5 (11.6)
Not performed	21 (48.8)		Calcineurin inhibitors (Ciclosporin A)	1 (2.3)
			B-cell targeted biologics (Rituximab, Ocrelizumab)	3 (7)
			Other biologics (Tocilizumab)	1 (2.3)
			Targeted therapies (Tyrosine kinase inhibitor)	1 (2.3)
			Pyrimidine synthesis inhibitor (Leflunomide)	1 (2.3)
Tetanus vaccine response		ANA titer	Negative	26 (60.5)
Positive	30 (69.8)		< 1/100	7 (16.3)
Negative	6 (14)		1/160	5 (11.6)
Not performed	7 (16.3)		> 1/320	3 (7)
Anti-CCP (+/–)	0/35(81.4)	Genetic Testing	Performed	26 (60.5)
RF (+/–)	1(2.3)/32(74.4)		CES	16 (61.5)
Anti-TG (+/–)	1(2.3)/31(72.1)		WES	5 (19.2)
Anti-TPO (+/–)	6(14)/34(79.1)		TNGS	4 (15.4)
Anti-dsDNA (+/–)	0/39(90.1)		SS	1 (3.8)

Abbreviations: Anti-TPO, anti–thyroid peroxidase antibody; Anti-CCP, anti–cyclic citrullinated peptide antibody; RF, rheumatoid factor; Anti-TG, anti-thyroglobulin antibody; Anti-dsDNA, anti–double-stranded DNA antibody; Anti-LKM, anti–liver kidney microsomal antibody; ASMA, anti–smooth muscle antibody; AMA, anti-mitochondrial antibody; ANA, antinuclear antibody; CES, clinical exome sequencing; WES, whole exome sequencing; TNGS, targeted next-generation sequencing; SS, Sanger sequencing

*Transferred from pediatric clinic

Genetic analysis was available for 26 patients. Pathogenic *TNFRSF13B* variants were identified in three patients; however, none fulfilled diagnostic criteria for CVID. Pathogenic variants in *MVK, FOXF1, TREX1*, and *C9* were also detected but showed no clear genotype–phenotype correlation. In addition, a truncating *ASXL1* p.Gln695* variant was detected in patient P1 with a variant allele frequency (VAF) of 31%, suggesting a somatically acquired mutation rather than a germline alteration. A frameshift variant in *CD19* (c.648_649delTG; p.Val217AlafsTer6) was identified in a consanguineous family during the evaluation of an affected child. The child was homozygous for the variant and exhibited a severe and classical CD19 deficiency phenotype, including recurrent bacterial infections, combined IgM and IgG deficiency, absent CD19 expression, preserved vaccine responses, p-ANCA positivity, hypocomplementemic immune-complex glomerulonephritis, nephrotic syndrome requiring dialysis, and ongoing IVIg therapy. Both parents were confirmed to be heterozygous carriers. The father, who was subsequently included in our cohort due to persistently reduced serum IgM levels, was heterozygous for the same variant and presented with isolated IgM deficiency and mild allergic manifestations without additional features of classical CD19 deficiency. The heterozygous mother was clinically asymptomatic and demonstrated a normal immunological evaluation. This intrafamilial distribution supports autosomal recessive inheritance of classical CD19 deficiency in the homozygous child; however, whether the isolated IgM reduction observed in the heterozygous father is related to carrier status or represents an independent finding remains uncertain. Several VUS, including *STAT6*, *CARD9*, and *DOCK8*, were detected in patients with suggestive but incomplete clinical phenotypes; functional studies are ongoing. Detailed genetic findings are provided in Supplementary Table 1.

Serum IgM levels were uniformly reduced, with median lowest and highest values of 25 and 27 mg/dL, respectively (Table 2). Elevated IgE levels were observed in 35.7% of patients. Reduced CD19⁺ B-cell and NK-cell counts were detected in 26.2% and 16.6% of patients, respectively. IgM memory B cells and naïve CD8⁺ T cells were decreased in 52.9% and 53.8% of patients, respectively, while increased TEM CD8⁺ T cells and elevated Tfh cells were observed in 38.5% and 45.5% of patients. Anemia-related parameters were frequently abnormal, with reduced creatinine in 48% and elevated serum amyloid A in 31%.

**Table 2:** Laboratory characteristics of the study cohort

	Reference ranges	*N*-Miss	Overall Median (Min–Max); *n*↓/*n*↑		Reference ranges	*N*-Miss	Overall Median (Min–Max); *n*↓/*n*↑
IgG(mg/dL)	767–1590	0	1064 (593–2640); 8↓/3↑	T cells (cells/µl)	700–2100	1	1466 (77-2612); 5↓/5↑
IgA(mg/dL)	61–356	0	149 (5-1138); 8↓/4↑	Helper T cells (cells/µl)	300–1400	1	865 (45-2106); 2↓/6↑
Lowest IgM(mg/dL)	37–286	0	25 (8–36)	Cytotoxic T cells (cells/µl)	200–900	1	449.5 (27-1097); 5↓/4↑
Highest IgM (mg/dL)	37–286	0	27 (16–47);	B cells (cells/µl)	100–500	1	164.5 (0-757); 11↓/3↑
IgE(kU/L)	< 100	1	25.95 (2-7600); 0↓/15↑	NK (cells/µl)	90–600	1	217 (10–663); 7↓/1↑
IgG1(mg/dL)	490–1140	3	672 (108–1170); 6↓/1↑	NKT (cells/µl)	10–150	37	187.5 (37–477);0↓/3↑
IgG2(mg/dL)	150–640	4	291 (112–650); 3↓/1↑	Naive B cells %	43-82.4	26	74.9 (1.5–91.3); 3↓/4↑
IgG3(mg/dL)	11–85	3	36.2 (11.4–106); 0↓/2↑	IgM memory cells %	7.2–30.8	26	5.6 (0-30.3); 9↓/0
IgG4(mg/dL)	3-200	2	45.8 (5.45–365); 0↓/4↑	Switched memory cells%	6.5–29.2	26	10.30 (1.1–46); 4↓/2↑
Leukocytes (×10³/mm³)	4.5–11	0	6.95 (2.59–15.81); 5↓/4↑	CD21 low cells%	0.8–7.7	26	3.5 (0.4-15.08); 1↓/3↑
Lymphocytes	650–2800	0	1980 (400–4530); 1↓/6↑	Transitional cells %	0.6–3.5	27	1.4 (0.07–4.5);3↓/1↑
Neutrophils (×10³/mm³)	1.8–7.7	0	4.53 (1.11–14.41); 3↓/3↑	Plazmoblast %	0.4–3.6	27	0.65 (0.02–4.5); 5↓/2↑
Monocytes (×10³/mm³)	0- 0.8	0	0.54 (0.22–1.33); 0↓/5↑	Naive CD4 + T cells %	14–67	30	34.2 (2.5–48.2); 2↓/0↑
Eosinophils (×10³/mm³)	0- 0.45	0	0.17 (0-1.18); 0/4↑	TCM CD4+ %	26–64	30	44.5 (32-57.5); 0↓/0↑
Erythrocytes (×10³/mm³)	4.52–5.9	0	4.82 (3.63–6.39); 15↓/1↑	TEM CD4+ %	5–30	30	20.8 (9-48.1); 0↓/3↑
Hemoglobin (g/dL)	13.5–17.5	0	13.8 (9.8–16.7); 15↓/1↑	TEMRACD4+ %	0–4	30	1.7 (0.2–42.1); 0↓/ 4↑
Hematocrit (%)	41.5–50.4	0	41.7 (30.6–51.5); 22↓/0	Naive CD8 + T cells %	25–73	30	24.2 (5.05–60.1); 7↓/0↑
Platelets (×10³/mm³)	150–450	0	268 (134–477); 1↓/1↑	TCM CD8+ %	6–40	30	11.5 (3.1–34.5);1↓/0
AST (U/L)	< 35	1	16.5 (10–47); 0↓/2↑	TEM CD8+ %	6–34	30	29.5 (19.5–54.7); 0↓/5↑
ALT (U/L)	< 45	1	19 (8–87); 0↓/2↑	TEMRA CD8%	5–33	30	17.6 (4.7–71.6); 1↓/4↑
ALP (U/L)	40–129	1	75.5 (41–197); 0↓/1↑	Tfh (CD4+) %	1.8–8.9	32	8.66 (3.6–14.4); 0↓/5↑
GGT (U/L)	< 55	1	22 (10–375); 0↓/4	γδ T cells %	1.0–11	31	3.65 (0.4–8.5): 1↓/0↑
Total protein (g/dL)	6.4–8.3	1	7.08 (5.93–8.3); 6↓/0	DNT %	0.4–2.5	31	0.45 (0.1–14.4);4↓/0
Albumin (g/dL)	3.5–5.2	1	4.43 (3.16–5.3); 1↓/2↑	Treg %	2.8-9.7	41	1.6 (0.1–3.1)
UREA (mg/dL)	10–50	1	29.5 (14–94); 0↓/1↑	TREC %		36	6.3 (3.9–26.7)
CREA (mg/dL)	0.7–1.3	1	0.83 (0.23–1.37); 13↓/1↑	C3 (mg/dL)	90–180	2	129 (34–208); 1↓ /2↑
LDH (U/L)	135–225	1	183.5 (131–380); 2↓/7↑	C4 (mg/dL)	10–40	2	28 (5–56); 2↓/6↑
β2M (µg/L)	651–2295	26	1802 (1142–2939); 0↓/5↑	Free Kappa Light Chain(mg/L)	6.7–22.4	22	16 (1.42–653); 5↓/5↑
SAA (mg/L)	< 6.4	18	6 (3–45); 0↓/12↑	Free Lambda Light Chain(mg/L)	8.3–27	22	17.6 (1.5–52.7); 6↓/4↑

Abbreviations: AST, aspartate aminotransferase; ALT, alanine aminotransferase; GGT, gamma-glutamyl transferase; ALP, alkaline phosphatase; LDH, lactate dehydrogenase, SAA, Serum amyloid A; β2M, Beta-2 Microglobulin; B cells, CD19⁺; CD21–/low B cells, CD21 low-expressing B cells; Cytotoxic T cells, CD3⁺CD8⁺; Double-Negative T Cells (DNT),: CD3⁺CD4⁻CD8⁻ T cells; Effector memory CD4⁺ T cells (TEMCD4⁺), CD4⁺CD45RA⁻CD27⁺; Effector memory CD8⁺ T cells (TEMCD8⁺), CCR7⁻CD8⁺CD45RA⁻CD27⁺; Follicular helper T cells (Tfh), CXCR5⁺PD-1⁺; Helper T cells, CD3⁺CD4⁺; IgM memory B cells,: CD27⁺IgM⁺CD19⁺; Naive B cells, CD45RA⁺IgD⁺CD27⁻; Naive CD4⁺ T cells, CD4⁺CD45RA⁺CD27⁺; Naive CD8⁺ T cells, CCR7⁺CD8⁺CD45RA⁺CD27⁺; NK cells, CD16⁺56⁺; NKT cells, CD3⁺CD16⁺56⁺; Plasmablasts, CD38⁺⁺CD138⁺; Switched memory B cells, CD45RA⁻IgD⁻CD27⁺; T cells, Total CD3⁺; Terminal effector memory CD4⁺ T cells (TEMRACD4⁺), CD4⁺CD45RA⁺CD27⁻; Terminal effector memory CD8⁺ T cells (TEMRACD8⁺), CCR7⁻CD8⁺CD45RA⁻CD27⁺; Transitional B cells, CD38⁺CD24⁺IgM⁺; γδ T cells, TCRγδ⁺;Treg, regulatory T cells (CD4⁺CD25⁺FOXP3⁺); TREC, T-cell receptor excision circles (marker of recent thymic emigrants / thymic output)

Values are presented as median (min–max); n↓ and n↑ indicate the number of patients below and above the laboratory reference range, respectively

Recurrent infections (58.1%) and allergic diseases (58.1%) were most frequent, followed by dermatologic (41.9%) and respiratory involvement (39.5%), while autoimmune conditions and osteoporosis were each observed in 37.2% of patients (Table 3). Infections predominantly involved the upper and lower respiratory tract, while allergic manifestations were heterogeneous, most commonly drug allergy, asthma, and allergic rhinitis. When ordered by median age at diagnosis, allergic diseases (23 years), respiratory involvement (32 years), and recurrent infections (36 years) represented the earliest manifestations, whereas dermatologic involvement (55 years) and autoimmune conditions (59 years) were diagnosed at older ages. Most allergic (96%), infectious (72%), respiratory (88.2%), dermatologic (72.2%), autoimmune (81.3%), neurological (71.4%), and endocrine manifestations (73.3%) were diagnosed before IEI recognition, whereas hepatobiliary involvement, lymphoproliferative disorders, osteoporosis, and malignancies showed a more heterogeneous timing, with approximately 43–81% diagnosed at or after IEI diagnosis. Notably, although six patients (12%) were identified through screening with incidentally detected low IgM levels, none exhibited completely isolated laboratory abnormalities. All individuals exhibited at least one documented clinical manifestation, including infectious, autoimmune, allergic, inflammatory, or organ-specific involvement.

**Table 3:** Comorbidities, age at diagnosis, timing in relation to IgM deficiency, and subtypes

Comorbidity	*n* (%)	Age at diagnosis, years (Median[range]; mean ± SD)	Onset timing vs. IEI: *n*(%)	Subtype: *n* (%)
Allergy	22 (51.2)	23 (4–65); 30.9 ± 19.3	Pre–primary Dx: 22 (100) At–primary Dx: 0 Post–primary Dx: 0	Allergic rhinitis:4 (17.4) Asthma: 5 (21.7) Asthma + atopic dermatitis: 1 (4.3) Asthma + rhinitis + drug allergy (COVID-19 vaccine): 1 (4.3) Allergic conjunctivitis with pruritus :1 (4.3) Drug allergy (various agents*)± urticaria: 7 (30.4) Contact dermatitis: 2 (8.7) Urticaria (isolated): 1 (4.3)
Respiratory involvement	17 (39.5)	32 (6–65); 35.1 ± 21.1	Pre–primary Dx: 15 (88.2) At–primary Dx: 2 (11.8) Post–primary Dx: 0	Asthma: 4 (23.5) Bronchiectasis: 4 (23.5) ILD: 2 (11.8) Asthma + bronchiectasis: 1 (5.9) Asthma + bronchiectasis + allergic rhinitis: 1 (5.9) OSAS + allergic rhinitis: 1 (5.9) COPD: 1 (5.9) COPD + ILD, PH: 1(5.9) OSAS + asthma + COPD + PH: 1 (5.9) Malignancy: 1 (5.9)
Recurrent infections	25 (58.1)	36 (10–71); 38.96 ± 21.3	Pre–primary Dx: 18 (72) At–primary Dx: 7 (28) Post–primary Dx: 0	ASYE ± LRTI: 18(72) UTI: 2 (8) Oral candidiasis: 2 (8) URTI + AGE: 2 (8) URTI + UTI + fungal skin inf.: 1 (4)
Nephrological involvement	3 (6.98)	42 (0–63); 35.0 ± 32.1	Pre–primary Dx: 2 (66.7) At–primary Dx: 1 (33.3) Post–primary Dx: 0	Alport syndrome: 1 (33.3) Horseshoe kidney: 1 (33.3) Nephrolithiasis: 1 (33.3)
Hepatobiliary involvement	10 (23.3)	43 (26–65); 45.4 ± 12.6	Pre–primary Dx: 3 (30) At–primary Dx: 5 (50) Post–primary Dx: 2 (20)	Hepatic steatosis: 7 (70) PBC + hepatic steatosis: 1 (10) Past HBV infection + hepatic steatosis: 1 (10) Gilbert syndrome: 1 (10)
Malignancy	7 (16.3)	50 (38–76); 54.4 ± 16.2	Pre–primary Dx: 4 (57.1) At–primary Dx: 0 Post–primary Dx: 3 (42.9)	DLBCL + FL: 1 (14.3) CML: 1 (14.3) Colon adenocarcinoma + cSCC:1 (14.3) NSCLC: 1 (14.3) Lymphoma (not otherwise specified): 1 (14.3) Metastatic NSCLC + Metastatic HSPC: 1 (14.3) PAC + subsequently diagnosed with MM: 1 (14.3)
Lymphoproliferation disorder	10 (23.3)	53 (23–75); 53.4 ± 14.4	Pre–primary Dx: 3 (33.3) At–primary Dx: 5 (55.6) Post–primary Dx: 1 (11.1)	Inguinal LAP:6 (60.0) Axillary LAP: 5 (50.0) Cervical LAP: 4 (40.0) Thoracic LAP:1 (10.0) Abdominal LAP: 1 (10.0) Splenomegaly: 2 (20.0)
Endocrine disorder	15 (34.9)	53 (41–70); 54.3 ± 10.0	Pre–primary Dx: 11 (73.3) At–primary Dx: 1(6.7) Post–primary Dx: 3 (20)	Type 2 DM: 7 (46.7) Type 2 DM+ hypothyroidism: 3 (20) Type 2 DM + Hashimoto thyroiditis: 2 (13.3) Hypothyroidism: 1 (6.7) Graves disease: 1 (6.7) Thyroid nodule: 1 (6.7)
Vitamin deficiency	14 (32.6)	54 (22–64); 49.0 ± 13.2	Pre–primary Dx: 6 (50) At–primary Dx: 1 (8.3) Post–primary Dx: 5 (41.7)	Iron deficiency: 8 (57.1) Vitamin B12 deficiency: 3 (21.4) Vitamin B12 + iron deficiency: 2 (14.3) Zinc + copper deficiency: 1 (7.1)
Neurological disorder	7 (16.3)	55 (17–70); 50.3 ± 17.2	Pre–primary Dx: 5 (71.4) At–primary Dx: 1 (14.3) Post–primary Dx: 1 (14.3)	Myasthenia gravis: 3 (42.9) Multiple sclerosis: 1 (14.3) Restless legs syndrome: 1 (14.3) Migraine: 1 (14.3) Vestibular neuritis: 1 (14.3)
Dermatologic involvement	18 (41.9)	55 (15–78); 51.9 ± 18.6	Pre–primary Dx: 13 (72.2) At–primary Dx: 5 (27.8) Post–primary Dx: 0	Eczema: 3 (16.,7) Chronic urticaria: 3 (16.7) Superficial fungal infections: 3(16.7) Seborrheic dermatitis: 2 (11.1) Calcinosis cutis: 1 (5.6) Necrobiosis lipoidica: 1 (5.6) Spongiotic dermatitis: 1 (5.6) Acne rosacea: 1 (5.6) Atopic dermatitis + wart, molluscum: 1 (5.6) Pemphigus: 1 (5.6) Bullous pemphigoid: 1 (5.6)
Gastrointestinal involvement	11 (25.6)	56.5 (22–79); 55.9 ± 14.8	Pre–primary Dx: 5 (45.5) At–primary Dx: 1(9.1) Post–primary Dx: 4(36.4)	Chronic gastritis: 7 (63.6) Autoimmune enteropathy: 1(9.1) Chronic diarrhea: 1 (9.1) Colon polyp: 1 (9.1) Tubular adenoma + chronic gastritis: 1 (9.1)
Osteoporosis	16 (37.2)	57.5 (21–78); 55.9 ± 14.4	Pre–primary Dx: 2 (13.3) At–primary Dx: 8 (53.3) Post–primary Dx: 5 (33.3)	-
Autoimmune condition	16 (37.2)	59 (25–78); 52.6 ± 15.5	Pre–primary Dx: 13 (81.3) At–primary Dx: 3 (18.8) Post–primary Dx: 0	Pemphigus: 1 (6.25) Bullous pemphigoid: 1 (6.25) Myasthenia gravis: 2 (12.5) Immune thrombocytopenia: 1 (6.25) Ankylosing spondylitis: 1 (6.25) Multiple sclerosis: 1 (6.25) Dermatomyositis: 1 (6.25) Systemic lupus erythematosus: 1 (6.25) Rheumatoid arthritis: 1 (6.25) Graves disease: 1 (6.25) Hashimoto thyroiditis: 2 (12.5) Hashimoto thyroiditis + CTD: 1 (6.25) Autoimmune enteropathy: 1 (6.25) PBC + Myasthenia gravis + panniculitis: 1 (6.25)

Abbreviations: OSAS, obstructive sleep apnea syndrome; COPD, chronic obstructive pulmonary disease; PH, pulmonary hypertension; NSIP, nonspecific interstitial pneumonia; ILD, interstitial lung disease; NSCLC, Non–small cell lung carcinoma; LAP, lymphadenopathy; PBC, Primary biliary cholangitis; DLBCL, Diffuse large B-cell lymphoma; cSCC: Cutaneous squamous cell carcinoma; CML, chronic myeloid leukemia; FL, follicular lymphoma; HSPC, hormone-sensitive prostate cancer; PAC, Prostate acinar adenocarcinoma; MM, Multiple myeloma; CTD, Connective tissue disease, ASYE: Acute suppurative otitis media; LRTI: Lower respiratory tract infection; UTI: Urinary tract infection; URTI: Upper respiratory tract infection; AGE: Acute gastroenteritis

*Includes antibiotics: fluoroquinolones, cotrimoxazole, beta-lactams; NSAIDs: aspirin, metamizole; contrast media

Across all IgM-based stratification strategies, most clinical features, immunological phenotypes, biochemical parameters, and clinical management variables—including administration of intravenous immunoglobulin replacement therapy (IgRT) and use of prophylactic antimicrobial therapy—were similarly distributed between groups, with no significant differences (Table 4). Lower IgM levels were associated with male sex, higher consanguinity rates, and lower total protein and albumin levels.

**Table 4:** Comparison of clinical and immunological characteristics of patients stratified by Highest IgM levels

Allergy *n*(%)	IgM ≤ 25 mg/dL (*n*:18)	IgM: 26–≤50 mg/dL (*n*:25)	*P* value	IgM ≤ 30 mg/dL (*n* = 25)	IgM 30<-<50 mg/dL (*n* = 18)	*P* value	IgM < 37 mg/dL (*n* = 32)	IgM 37≤-≤50 mg/dL (*n* = 11)	*P* value
	8 (44.4%)	14 (56%)	0.455	14 (56%)	8 (44.4%)	0.455	18 (56.3%)	4 (36.4%)	0.255
Respiratory involvement n(%)	8 (44.4%)	9 (36%)	0.576	11 (44%)	6 (33.3%)	0.480	13 (40.6%)	4 (36.4%)	> 0.99
Recurrent infections	11(61.15%)	14 (56%)	0.738	15 (60%)	10 (55.6%)	0.771	18 (56.3%)	7 (63.6%)	0.736
Nephrological involvement n(%)	1 (5.6%)	2 (8%)	> 0.99	2(8%)	1 (5.6%)	> 0.99	3 (9.4%)	0 (0.0%)	0.558
Hepatobiliary involvement n(%)	4 (22.2%)	6(24%)	> 0.99	6 (24%)	4 (22.2%)	> 0.99	7 (21.9%)	3 (27.3%)	0.698
Malignancy n(%)	4 (22.2%)	3(12%)	0.427	5 (20%)	2 (11.1%)	0.680	5 (15.6%)	2 (18.2%)	> 0.99
Lymphoproliferation disorder n(%)	6 (33.3%)	4 (16%)	0.275	7 (28%)	3 (16.7)	0.480	8 (25%)	2 (18.2%)	> 0.99
Endocrine disorder n(%)	4 (22.2%)	11 (44%)	0.139	7 (28%)	8 (44.4%)	0.264	12 (37.5%)	3 (27.3%)	0.719
Vitamin deficiency n(%)	6 (33.3%)	4 (28.6%)	> 0.99	-	-	-	-	-	-
Neurological disorder n(%)	1 (5.6%)	6 (24%)	0.209	3 (12%)	4 (22.2%)	0.427	3 (9.4%)	4 (36.4%)	0.058
Dermatologic involvement n(%)	8 (44.4%)	10 (40%)	0.771	12 (48%)	6 (33.3%)	0.336	14 (43.85)	4 (36.4%)	0.736
Gastrointestinal involvement n(%)	4 (22.2%)	7 (28%)	0.736	7 (28%)	4 (22.2%)	0.736	9 (28.1%)	2 (18.2%)	0.698
Osteoporosis	8 (44.4%)	8 (32%)	0.405	11 (44%)	5 (27.8%)	0.278	13 (40.6%)	3 (27.3%)	0.494
Autoimmune condition n(%)	6 (33.3%)	10 (40%)	0.655	10 (40%)	6 (33.3%)	0.655	12 (37.5%)	4 (36.4%)	> 0.99
Gender n(%): Male Female	12 (66.7%) 6 (33.3%)	9 (36%) 16(64%)	0.047	14 (56%) 11 (44%)	7 (38.9%) 11 (61.1%)	0.268	15 (46.9%) 17 (53.1%)	6 (54.5%) 5 (45.5)	0.661
Consanguinity n(%)	7 (38.9%)	3 (12%)	0.017	8 (32%)	2 (11.1%)	0.016	10 (31.3%)	0 (0.0%)	0.19
Isohemagglutinin status n(%): Positive Negative	2 (11.1%) 6 (33.3%)	8 (32%) 13 (52%)	0.675	3 (12.5%) 11 (45.8%)	7 (41.2%) 8 (47.1%)	0.245	6 (19.4%) 15 (48.4%)	4 (40%) 4 (40%)	0.390
Tetanus vaccine response n(%): Positive Negative	14 (77.8%) 1 (5.6%)	16 (64%) 5 (20%)	0.367	17 (68%) 4 (16%)	13 (72.2%) 2 (11.1%)	0.689	22 (68.8%) 5 (15.6%)	8 (72.7%) 1 (9.1%)	0.676
Pneumococcal vaccine response n(%): Positive Negative	9 (50%) 1 (5.6%)	11 (44%) 1 (4%)	> 0.99	12 (48%) 1 (4%)	8 (44.4%) 1 (5.6%)	> 0.99	15 (46.9%) 2 (6.3%)	5 (45.5%) 0 (0.0%)	> 0.99
Total protein (g/dL) Median (Min–Max)	6.4 (6.11–7.25)	7.2 (6.79–7.68)	0.002	7.13 (6.11–7.67)	6.99 (6.79–7.68)	0.151	7.08 (5.93–8.3)	7.15 (6.4–7.68)	0.374
Albumin (g/dL) Median (Min–Max)	4.06 (3.75–4.72)	4.56 (3.67–4.63)	0,038	4.18 (3.75–4.72)	4.42 (3.67–4.63)	0.148	4.26 (3.16–5.3)	4.53 (4.08–4.98)	0.213
Naive B cells median (min–max, cells/µL) (%)	77.4 (62.3–88.4)	38.3 (1.5–66.8)	0.027	68.9 (1.5–88.4)	55.5 (30.2–66.8)	0.763	71.15 (1.5–91.3)	74.9 (30.2–78.9)	0.705
Naive CD4 median (min–max, cells/µL) (%)	29(14-38.8)	37 (3.1–47.7)	0.749	34.2 (14-47.7)	19.3 (3.1–42.1)	0.090	37.65 (14-48.2)	2.8 (2.5–3.1)	0.028*
TCMCD4 median (min–max, cells/µL) (%)	45.9 (33.1–54.3)	44.55 (33.9–57.50)	0.475	44 (33.1–54.3)	56.5 (44.6–57.5)	0.043*	44 (32-57.5)	44.6 (44.6–44.6) ^1^	0.789

*Due to small subgroup sizes (e.g., *n* = 2 for naive CD4⁺ T cells in the IgM ≥ 37 mg/dL group; *n* = 3–4 in other subsets), statistical power is limited and results should be interpreted with caution

According to the 2019 ESID Registry working definitions, 7 patients (16.3%) fulfilled the diagnostic criteria for SIgMD. Among the 25 patients with clinically significant infections, 18 did not meet these criteria due to concomitant immunoglobulin abnormalities or incomplete evaluation of vaccine responses, including IgA deficiency (*n* = 4), IgG1 deficiency (*n* = 3), and IgG2 deficiency (*n* = 4), as well as missing or impaired responses to tetanus (not performed *n* = 4, negative *n* = 4) and PPSV23 vaccination (not performed *n* = 11, insufficient response *n* = 2). According to the ESID clinical criteria, 35 patients (81.4%) were classified as unPAD, presenting with at least one of the following features: recurrent infections, autoimmune manifestations, or lymphoproliferation. One patient did not meet the criteria for either category.”

In logistic regression analyses, serum IgM levels were not significantly associated with most clinical, immunological, or treatment-related outcomes (all *p* > 0.05). Non-significant trends were observed for neurological disorders (OR = 1.10, 95% CI: 0.99–1.23, *p* = 0.085) and isohemagglutinin positivity (OR = 1.10, 95% CI: 0.98–1.24, *p* = 0.111). Parental consanguinity was the only variable independently associated with serum IgM levels (OR = 0.81, 95% CI: 0.68–0.97, *p* = 0.018) (Supplementary Table 2). ROC curve analyses demonstrated limited discriminatory performance of serum IgM across evaluated outcomes, with AUC values ranging from 0.286 to 0.574. The highest AUC values were observed for lymphoproliferation (AUC = 0.574) and malignancy (AUC = 0.573). Isohemagglutinin positivity showed a numerically higher AUC (0.679; 95% CI: 0.466–0.892); however, the wide confidence interval indicates considerable uncertainty in discriminatory performance (Supplementary Table 3). Spearman correlation analysis showed positive correlations between serum IgM and total protein (rho = 0.356, *p* = 0.021) and albumin (rho = 0.336, *p* = 0.030), with no significant correlations observed for other immunological, hematological, or biochemical parameters.

Overall, IgRT exposure was limited, and most patients were IgRT-naïve at the time of evaluation; prior IgRT use was largely related to autoimmune or inflammatory indications before referral, and most of these patients (6/7) were lost to follow-up. During follow-up at our center, IgRT was initiated in three patients due to recurrent infections accompanied by significant immunological abnormalities. All three patients demonstrated adequate tetanus vaccine responses. One patient with myasthenia gravis and primary biliary cirrhosis had frequent upper respiratory tract infections and marked B-cell abnormalities, with an adequate PPSV23 response but low isohemagglutinin titers. A second patient with widespread eczema and recurrent viral skin infections showed impaired pneumococcal vaccine responses despite adequate isohemagglutinin titers. The third patient, who had persistently low IgA and IgM levels with reduced IgG4, experienced recurrent gastroenteritis and upper respiratory infections; PPSV23 response was not available, while isohemagglutinin titers were adequate. Following IgRT initiation, all three patients experienced a clear reduction in infection frequency and clinical improvement. In addition, prophylactic antimicrobial therapy was administered in 12 patients (27.9%) and was associated with improved infection control.

During follow-up, pathogenic microorganisms were identified in 18 patients (41.9%) from various clinical specimens obtained during infectious episodes. The most frequently detected bacterial pathogens were *Staphylococcus aureus* (*n* = 4), *Haemophilus influenzae non–type b* (*n* = 2), and *Pseudomonas aeruginosa* (*n* = 2), with single detections of *Streptococcus pneumoniae, Streptococcus agalactiae, Escherichia coli*, and *Campylobacter jejuni*. Viral agents included *SARS-CoV-2* (*n* = 3; one severe case), *Epstein–Barr virus* (*n* = 2), *norovirus type 2* (*n* = 1), and *herpes simplex virus* (*n* = 1). Fungal and parasitic isolates comprised *Candida albicans* (*n* = 3), *Candida parapsilosis* (*n* = 1), *Candida tropicalis* (*n* = 1), *Candida glabrata* (*n* = 1), *Aspergillus fumigatus complex* (*n* = 1), *Trichophyton rubrum* (*n* = 1), *Blastocystis spp.* cysts (*n* = 1), *Cryptosporidium spp.* oocysts (*n* = 3), and *Demodex folliculorum* (*n* = 1).

Allergy testing (skin prick and/or specific IgE) was available in 13 patients (30.2%), of whom 7 (53.8%) showed at least one positive sensitization. The most frequently identified sensitizations were to *Dermatophagoides farinae* (*n* = 5) and *Dermatophagoides pteronyssinus* (*n* = 5). Other inhalant allergens included olive pollen (*Olea europaea*, *n* = 3), rye (*Secale cereale*, *n* = 1), mugwort (*Artemisia vulgaris*, *n* = 1), plantain (*n* = 1), and mixed grass pollens (*n* = 2). Sensitization to mold (*Aspergillus* spp.) was detected in one patient.

## Discussion

In this adult IEI cohort, persistently reduced serum IgM levels were identified in approximately one in nine patients (11.4%), highlighting that low IgM in adulthood is not an exceptional finding in tertiary immunology practice. Importantly, only a minority of patients (16.3%) fulfilled strict ESID criteria for SIgMD, whereas the majority were classified as unPAD, underscoring the heterogeneity of low IgM–associated phenotypes in adulthood. Clinically, infectious, allergic, autoimmune, and lymphoproliferative manifestations frequently overlapped. Serum IgM concentration alone demonstrated limited ability to discriminate clinical phenotypes or predict disease severity. Collectively, these findings indicate that reduced IgM in adults should be interpreted within a broader clinical and immunological context rather than as a standalone diagnostic marker.

The proportion of adults with persistently reduced serum IgM levels observed in our cohort appears higher than prevalence estimates previously reported for selective IgM deficiency in both population-based studies and immunodeficiency clinic cohorts [11]. This difference is likely attributable, at least in part, to referral bias inherent to tertiary care centers, where patients with complex clinical courses, multisystem involvement, and atypical immunological phenotypes are more frequently evaluated. In addition, unlike studies restricted to “complete” SIgMD, our cohort included a broader spectrum of adults with sustained IgM reduction [12]. Current diagnostic definitions of SIgMD are largely based on exclusion criteria and may therefore fail to capture a subset of affected individuals, particularly adults presenting with overlapping or atypical clinical phenotypes. Consistent with this notion, most patients in our cohort were referred for consultation because of incidentally detected low immunoglobulin levels rather than classical IEI-related clinical manifestations. Despite this referral pattern, we did not identify a truly asymptomatic subgroup among individuals with persistently reduced IgM levels. Although a small proportion of patients were initially detected through screening with incidentally identified low IgM levels, all individuals ultimately exhibited at least one infectious, allergic, autoimmune, or organ-specific clinical manifestation. Similar observations have been reported in other referral-based cohorts. In a multicenter study, the vast majority of patients with selective IgM deficiency were symptomatic (≈ 97%), reflecting the predominance of clinically referred individuals evaluated in specialized immunology settings [21]. Taken together, the relatively high frequency of reduced IgM observed in our cohort likely reflects both tertiary referral bias and the broader inclusion of patients with persistently reduced IgM levels based on our predefined cut-off (< 50 mg/dL).

Recurrent infections represent the most commonly reported clinical manifestation of selective IgM deficiency and were also the most frequent presentation in our cohort [16, 18]. However, growing evidence indicates that selective or isolated IgM deficiency should not be considered solely a defect of antimicrobial defense but rather a broader state of immune dysregulation [29]. Consistent with this concept, studies in adult cohorts have reported substantial rates of allergic and autoimmune manifestations in IgM deficiency, with allergic diseases occurring in approximately one-third of patients and autoimmune disorders in up to 40–45% [16, 18]. In line with these observations, allergic diseases were identified in 58.1% of patients in our cohort and included heterogeneous phenotypes such as allergic rhinitis, asthma, drug allergy, urticaria, and contact dermatitis. Autoimmune conditions were also common (37.2%) and encompassed a wide spectrum of organ-specific and systemic diseases, including autoimmune thyroid disease, myasthenia gravis, systemic lupus erythematosus, dermatomyositis, and autoimmune enteropathy. Allergic manifestations tended to occur earlier in life, whereas autoimmune diseases were diagnosed later in adulthood, suggesting a possible temporal evolution of immune dysregulation in individuals with IgM deficiency. Distinct age-related patterns have been described in previous studies, with pediatric cohorts predominantly characterized by infectious phenotypes, whereas adult patients more often exhibit overlapping infectious, allergic, and autoimmune features [17]. These observations may reflect the dynamic evolution of immune dysregulation associated with IgM deficiency across the lifespan.

The increased susceptibility to allergic and autoimmune manifestations observed in IgM deficiency may be explained by several underlying immunological mechanisms. Natural IgM plays an important regulatory role in immune tolerance by facilitating the non-inflammatory clearance of apoptotic cells and modified self-antigens [30, 31]. When IgM levels are reduced, apoptotic debris and altered self-structures may persist longer in tissues and become available for uptake by antigen-presenting cells, thereby promoting immune activation rather than tolerogenic clearance [31]. In addition to this housekeeping function, IgM contributes to the regulation of B-cell tolerance during early B-cell development [32, 33]. Experimental studies have demonstrated that the absence of secreted IgM is associated with impaired central tolerance and expansion of autoreactive B-cell clones in the peripheral repertoire, leading to increased autoantibody production [34]. Furthermore, serum IgM deficiency has been associated with dysregulated antibody responses characterized by reduced responses to foreign antigens but increased spontaneous IgG autoantibody production [35]. Antibody deficiencies are also frequently accompanied by alterations in mucosal barrier integrity and microbial homeostasis, which may increase antigen exposure and promote Th2-skewed immune responses [26, 36, 37]. Collectively, disruption of these regulatory pathways may predispose individuals with IgM deficiency to both autoimmune and allergic manifestations.

Beyond clinical heterogeneity, our cohort demonstrated a functional dissociation between naturally occurring IgM-mediated humoral immunity and antigen-induced IgG responses. Vaccine responses were largely preserved, with adequate responses observed in 90.9% of patients following PPSV23 vaccination and in 83.3% after tetanus vaccination, whereas naturally occurring IgM-mediated immunity was frequently impaired, as reflected by reduced isohemagglutinin titers in 66% of patients. Similar dissociation between preserved vaccine responses and impaired natural IgM-mediated immunity has been reported in other adult SIgMD cohorts [7, 16, 38]. Protein antigens such as tetanus toxoid induce primarily T-cell–dependent responses through germinal center reactions, class-switch recombination, and memory B-cell formation, whereas polysaccharide vaccines such as PPSV23 predominantly stimulate T-cell–independent type-2 responses mediated by marginal zone and B1 B cells, although partial overlap between these pathways exists [39–41]. Clinically, defects in T-cell–independent polysaccharide responses are typically associated with susceptibility to encapsulated bacteria, whereas impairments in T-cell–dependent immunity more often predispose to viral and intracellular pathogens [42, 43]. Natural IgM antibodies, including isohemagglutinins, constitute an important component of innate-like humoral immunity and contribute to early host defense through complement activation, opsonization, and facilitation of antigen handling [44, 45]. Reduced isohemagglutinin titers in our cohort may therefore reflect impairment of this natural IgM axis despite largely preserved antigen-induced IgG responses. Consequently, even when vaccine-induced IgG levels appear adequate, early IgM-mediated immune defense—particularly at mucosal surfaces—may remain compromised and contribute to susceptibility to infections with encapsulated bacteria such as Streptococcus pneumoniae.

Respiratory infections were common in our cohort, although microbiological findings revealed a heterogeneous pathogen spectrum rather than a predominance of classical encapsulated bacteria. This observation may appear discordant with the largely preserved vaccine responses. However, standard ELISA-based assays primarily quantify serum IgG levels and may not fully reflect antibody functionality or mucosal immune protection. Reduced natural IgM levels may therefore influence the early coordination and overall quality of humoral immune responses even when conventional vaccine-induced IgG titers appear preserved [44, 46]. Notably, a subset of patients also exhibited concomitant IgA deficiency, which may further compromise mucosal barrier immunity and synergize with impaired IgM-dependent early defense, thereby amplifying susceptibility to recurrent respiratory and gastrointestinal infections despite preserved serum IgG vaccine responses [47, 48]. Additional factors, including airway structural disease, immunosuppressive therapies, and broader immune dysregulation, may further contribute to infection susceptibility and may partly explain the clinical benefit of immunoglobulin replacement therapy in selected patients. Consistent with the heterogeneous SIgMD literature, abnormalities in non-IgM immunoglobulin levels and lymphocyte subsets were variable [7, 29, 38]. Reduced IgM memory B cells and naïve CD8⁺ T cells emerged as the most consistent immunophenotypic abnormalities, accompanied in subsets of patients by reductions in CD19⁺ B cells and NK cells, expansion of TEM CD8⁺ T cells, and elevated IgE levels. Collectively, these findings suggest that reduced IgM levels in adulthood frequently occur within a broader context of immune dysregulation rather than representing an isolated quantitative antibody defect.

In the present study, we applied three complementary IgM-based stratification strategies—analyzer-derived cut-offs (≤ 25 mg/dL vs. >25–<50 mg/dL), literature-based thresholds (< 30 mg/dL vs. ≥30–<50 mg/dL), and institutional reference limits (< 37 mg/dL vs. ≥37–<50 mg/dL)—to evaluate whether different IgM levels identify clinically meaningful subgroups. Across all approaches, clinical manifestations, immunological phenotypes, antibody response to vaccines (and isohemagglutinins), biochemical parameters, and management-related variables were similarly distributed between groups, and multivariable regression and ROC analyses demonstrated limited discriminatory performance of serum IgM across evaluated outcomes. Comparable findings have been reported in previous cohorts. In a study of 62 adults with selective IgM deficiency stratified by IgM levels (≤ 30 mg/dL vs. >30 mg/dL), no differences were observed in IgG subclass deficiency, lymphocyte subsets, pneumococcal vaccine responses, or response to immunoglobulin replacement therapy [16]. Similarly, a multicenter cohort of 75 patients comparing fixed diagnostic cut-offs with population-based reference thresholds (< 2 SD or <3rd percentile) demonstrated largely comparable clinical and immunological profiles across groups [21]. Across cohorts, lower quantitative IgM levels did not predict a distinct or more severe clinical or immunological phenotype, supporting the concept that serum IgM concentration alone has limited predictive value for clinical risk stratification. Given that several AUC values approached or fell below the threshold of random discrimination (AUC ≈ 0.50), these ROC-derived cut-off estimates should be interpreted with caution.

From a pathogenetic and genetic standpoint, our findings further support the concept that selective or predominant IgM deficiency represents a heterogeneous and multifactorial immune phenotype rather than a single-gene disorder. In line with previous reports, no uniform inheritance pattern or causative mutation could be identified in our cohort [4, 21]. Although pathogenic *TNFRSF13B* variants were detected in three patients, none fulfilled diagnostic criteria for CVID. Heterozygous *TNFRSF13B* variants are increasingly recognized as susceptibility alleles with variable penetrance rather than fully penetrant monogenic causes of primary antibody deficiency, and their clinical expression appears to be influenced by additional genetic and environmental modifiers. Recent large-cohort analyses of *TNFRSF13B* mutation carriers further support this model, demonstrating substantial phenotypic variability and incomplete penetrance, particularly in monoallelic carriers [49]. Other pathogenic variants, including *MVK, FOXF1, TREX1,* and *C9*, showed no clear genotype–phenotype concordance in our cohort. In addition, a truncating *ASXL1* p.Gln695* variant identified in patient P1 showed a VAF of 31%, suggesting a somatically acquired alteration rather than a germline variant. Given that truncating variants in *ASXL1* are well-recognized drivers of age-related clonal hematopoiesis (CHIP), this finding was interpreted as a presumed somatic event related to clonal hematopoiesis rather than an inherited immune-related genetic defect.

In one patient harboring two *MVK* variants classified as pathogenic and another patient with a single pathogenic *MVK* variant, a classical mevalonate kinase deficiency phenotype—such as recurrent febrile episodes or systemic autoinflammatory manifestations—was not observed. Although these variants fulfill ACMG criteria for pathogenicity, clinical expression consistent with mevalonate kinase deficiency was not evident in our cohort. These observations further illustrate the complexity of genotype–phenotype correlations and the variability of clinical expression in adult IEI. Notably, a *CD19* frameshift variant was identified in one consanguineous family in which the affected child was homozygous and exhibited a classical severe CD19 deficiency phenotype. Both parents were heterozygous carriers; however, only the father demonstrated isolated low serum IgM and was therefore included in our cohort. While the homozygous state was clearly associated with a typical autosomal recessive phenotype, the clinical significance of isolated IgM reduction in the heterozygous father remains uncertain. The absence of immunological abnormalities in the heterozygous mother further underscores the variability of immune findings among carriers and cautions against attributing causality to a single heterozygous variant in isolation. In addition, several variants of uncertain significance (*STAT6*,* CARD9*,* DOCK8*) were identified in patients with partial or atypical clinical features. Overall, these findings underscore the need for integrative genomic and functional approaches to clarify underlying mechanisms.

Given the broad clinical spectrum of predominant IgM deficiency, patient management should not be limited to infection control but should adopt a multidisciplinary approach addressing commonly coexisting allergic, autoimmune, and dermatologic manifestations, as well as abnormal lymphoproliferation and malignancy. In this context, no standardized treatment strategy currently exists, and our findings support that IgRT should not be routinely indicated, but rather reserved for selected patients with clinically significant recurrent infections accompanied by objective evidence of humoral dysfunction, such as impaired vaccine responses or combined immunoglobulin abnormalities [4, 16, 50]. IgRT was initiated only in a small subset of patients with demonstrable functional immune impairment and was consistently associated with a reduction in infection frequency and clear clinical benefit. Importantly, preserved vaccine-induced IgG responses in a substantial proportion of patients support vaccination strategies and prophylactic antimicrobial therapy as first-line preventive measures in most cases. Collectively, these findings support a phenotype-driven, individualized management approach rather than routine immunoglobulin replacement based solely on low serum IgM levels.

Several limitations of this study should be acknowledged. First, the relatively modest sample size and single-center design may have limited the statistical power to detect subtle associations between serum IgM levels and specific clinical outcomes, particularly in subgroup analyses. Additionally, the small number of events for several outcomes further restricts the interpretability of subgroup comparisons and predictive analyses, and therefore the ROC analyses should be considered exploratory rather than definitive. Furthermore, as this study was conducted in a tertiary referral center, referral bias is likely, and asymptomatic individuals with isolated low IgM may be underrepresented. Second, heterogeneity in clinical presentation, follow-up duration, and availability of immunological investigations—including incomplete vaccine response data due to real-world logistical and financial constraints—may have introduced variability and reduced comparability across patients. Moreover, not all laboratory and immunophenotyping assessments were available for every patient, which may limit direct comparability across individuals. In addition, genetic testing was not uniformly performed using the same platform in all patients, reflecting the retrospective design and evolving availability of genetic technologies over the study period. Third, despite the application of multiple IgM-based stratification strategies and complementary ROC analyses, the retrospective design precluded standardized longitudinal assessment of functional immune responses. Nevertheless, the study also has important strengths. It represents one of the more comprehensive adult cohorts with persistently reduced serum IgM, integrating detailed clinical phenotyping, functional antibody responses, extended lymphocyte subset analysis, genetic evaluation, and multiple statistical approaches. Importantly, by analyzing IgM both as a continuous variable and across different clinically relevant thresholds, our study provides a nuanced and methodologically robust assessment of the limitations of serum IgM concentration as a standalone biomarker.

Taken together, our findings support the interpretation that reduced serum IgM in adults is more appropriately viewed as a marker of broader immune dysregulation rather than a discrete immunodeficiency entity. Reliance on infection history or absolute IgM thresholds alone appears insufficient to capture the full clinical spectrum associated with IgM deficiency [29]. From a clinical standpoint, patients with low IgM—particularly those with profound or persistently reduced levels—may benefit from longitudinal follow-up integrating functional antibody assessment, detailed B- and T-cell immunophenotyping, and surveillance for non-infectious complications such as autoimmunity, cytopenias, allergy, and lymphoproliferation. Importantly, the absence of robust predictive cut-off values underscores that IgM concentration should not be used in isolation to guide therapeutic decisions, including immunoglobulin replacement therapy, but rather as part of a comprehensive, individualized risk assessment. Future multicenter, prospective studies incorporating standardized functional assays and systematic genetic testing are needed to refine risk stratification, clarify disease trajectories, and inform evolving diagnostic and management algorithms for selective or predominant IgM deficiency across the lifespan.

## Supplementary Information

Below is the link to the electronic supplementary material.

Supplementary File 1 (DOCX 48.5 KB)

Supplementary File 2 (DOCX 46.0 KB)

## Data Availability

Availability of data and materials: The authors confirm that the data supporting the findings of this study are available within the article and/or its supplementary materials. If you have questions regarding the data, contact the corresponding author.
