# Supplementary material for: Low IgM Levels in Adult IEI: Classification Challenges and Clinical Implications
Source: J Clin Immunol. 2026 Apr 14;46(1):54. doi: 10.1007/s10875-026-02017-x (PMC13186790; doi:10.1007/s10875-026-02017-x)
Supplement: Supplementary file 1 — (DOCX 48.5 KB) [file 10875_2026_2017_MOESM1_ESM.docx]

**Supplementary Table 1. Summary of Genetic Analyses and Identified Variants**

| **Patient** | **Key clinical features** | **Method** | ***Gene*** | **Transcript** | **Zygosity** | **cDNA** | **Protein** | **Prevalence** | **ACMG** |
| --- | --- | --- | --- | --- | --- | --- | --- | --- | --- |
| P1 | Recurrent infections,  Bullous pemphigoid,  Lung carcinoma | CES | *TNFRSF13B*  *ASXL1* | *NM_012452.3*  *NM_015338.6* | Het  Het | c.204dup  c.2083C>T | p.Leu69Thrfs*12  p.Gln695* | Pd  Novel | Pathogenic  Likely pathogenic  (PVS1, PM2) |
| P2 | Recurrent infections, Chronic urticaria,  Bronchiectasis | CES | *FOXF1* | *NM_001451.3* | Het | c.1070A>T | p.His357Leu | Pd | Pathogenic |
| P3 | Recurrent infections,  Allergic rhinitis,  Chronic myeloid leukemia | CES | *CCDC40*  *PLCG2*  *CFTR* | *ENST00000374877*  *NM_002661.5*  *NM_000492.4* | Hom  Het  Het | c.2852_2883del  c.3793T>A  c.3454G>C | p.Gln951ArgfsTer132  p.Ser1265Thr  p.Asp1152His | Novel  Novel  Pd | Likely pathogenic (PVS1, PM2)  VUS (PM2, BP4)  Pathogenic |
| P4 | Lymphadenopathy,  Alport syndrome | CES | *COL4A4*  *COL4A4* | *NM_000092.5*  *NM_000092.5* |  | c.2320G>C  c.4394G>A | p.Gly774Arg  p.Gly1465Asp | Pd  Pd | Pathogenic  Pathogenic |
| P6 | Chronic urticaria,  Drug allergy,  Triptaz; 5,99µg/L(N) | TNGS | *KIT* | *NM_000222.3* | Het | c.2410C>T | p.Arg804Trp | Novel | VUS (PM2, PM1) |
| P12 | Oral kandidiyazis | WES | *PTPN22* | *NM_015967.7* | Het | c.1411T>A | p.Tyr471Asn | Novel | VUS (PM2, BP4) |
| P13 | Recurrent infections,  Lymphadenopathy, Evans syndrome,  Deep vein thrombosis | TNGS | *TREX1* | *NM_033629.6* | Het | c.-26-1G>A |  | Pd | Pathogenic |
| P15 | Recurrent infections | TNGS |  |  |  |  |  |  | Normal |
| P16 | Recurrent infections,  Graves’ disease,  Lymphadenopathy  Allergic rhinitis | CES |  |  |  |  |  |  | Normal |
| P17 | Recurrent infections | TNGS |  |  |  |  |  |  | Normal |
| P19 | Lymphadenopath  Colon cancer,  Allergic rhinitis | CES | *MAPK8IP1* | *NM_005456.4* | Het | c.1252T>C | p.Ser418Pro | Novel | VUS (PM2) |
| P20 | Recurrent infections  Asthma | CES |  |  |  |  |  |  | Normal |
| P25 | Asthma,  Allergic rhiniti  Congenital dysfibrinogenemia | WES | *TNFRSF13B*  *FGA* | *NM_012452.3*  *NM_021871.4* | Het  Het | c.310T>C  c.167C>G | p.Cys104Arg  p.Ser56Cys | Pd  Novel | Pathogenic  VUS (PM2) |
| P26 | Recurrent infections,  Asthma, Allergic rhinitis  Lymphadenopathy | CES | *CFTR*  *CFTR* | *NM_000492.4*  *NM_000492.4* | Het  Het | c.5A>C  c.3154T>G | p.Gln2Pro  p.Phe1052Val | Novel  Novel | VUS (PM2, PP2, PP3)  Likely pathogenic (PM1,PP2,PM2,PP3,PP5) |
| P27 | Recurrent infections,  Chronic diarrhea,  Drug allergy | CES |  |  |  |  |  |  | Normal |
| P29 | Non-Hodgkin lymphoma,  Contact dermatitis | CES |  |  |  |  |  |  | Normal |
| P30 | Recurrent infections,  Lung carcinoma,  Drug allergy,  Allergic rhinitis,  Oral ulcers | WES | *ELANE*  *MVK*  *MVK* | *NM_001972.4*  *NM_000431.4*  *NM_000431.4* | Het  Het  Het | c.781G>C  c.803T>C  c.1129G>A | p.Asp261His  p.Ile268Thr  p.Val377Ile | Novel  Pd  Pd | VUS (PM2,PP2,BP4)  Pathogenic  Pathogenic |
| P31 | Recurrent infections,  Asthma,  Atopic dermatitisc,  Lymphadenopathy,  Horseshoe kidney | WES | *TNFRSF13B*  *MVK* | *NM_012452.3*  *NM_000431.4* | Het  Het | c.418G>A  c.928G>A | p.Glu140Lys  p.Val310Met | Pd  Pd | Pathogenic  Pathogenic |
| P32 | Lymphadenopathy,  Drug allergy | CES | *MEFV*  *MEFV*  *C9* | *ENST00000219596*  *ENST00000219596*  *ENST00000263408.5* | Het  Het  Het | c.2080A>G  c.442G>C  c.162C>A | p.Met694Val  p.Glu148Gln  p.Cys54Ter | Pd  Pd  Pd | Pathogenic  Pathogenic  Pathogenic |
| P33 | Recurrent infections,  Nephrolithiasis,  Myasthenia gravis,  Panniculitis,  Primary biliary cholangitis | CES | *FOXN1*  *ABCC8* | *NM_001369369.1*  *NM_000352.6* | Het  Het | c.1154T>C  c.1252T>C | p.Ile385Thr  p.Cys418Arg) | Novel  Pd | VUS (PM2)  Likely benign |
| P35 | Allergic rhinitis | SS | CD19 | *NM_001770.6* | Het | c.648_49delTG | p.(Val217Alafs*6) | Novel | Likely pathogenic  (PVS1, PM2) |
| P36 | Contact dermatitis,  Pruritic plaques,  IgE ~10,000 IU/mL, Eosinophilia | WES | *STAT6* | *NM_003153.5* | Het | DEL: chr12:57100681-57100716 |  | Novel | VUS |
| P38 | Multiple sclerosis, Family history of cirrhosis | CES | *PRX*  *CASP10* | *NM_181882.3*  *NM_032977.4* | Het  Het | c.122G>A  DEL: chr2:201203439-201209817 | p.Gly41Glu | Novel  Novel | VUS (PM2)  VUS |
| P39 | Recurrent infections,  Lymphadenopathy,  Prostate cancer,  Allergic rhinitis | CES | *NFKB2* | *NM_001322934.2* | Het | c.1343C>T | p.Ala448Val | Novel | VUS (PM2, PP2, BP4) |
| P40 | Oral Candidiasis,  Seborrheic dermatitis | CES | *CARD9* | *NM_052813.5* | Het | c.1434+7C>A |  | Novel | VUS (PM2,BP4) |
| P43 | Recurrent infections,  Lymphadenopathy  Spongiotic dermatitis, Stasis dermatitis,  EBV viremia | CES | *DOCK8*  *KRT6C* | *NM_203447.4*  *NM_173086.5* | Hom  Het | c.6064A>G  c.1156C>T | p.Met2022Val  p.Arg386Cys | Novel  Novel | VUS (PM2, BP6)  VSU (PP3, PM2) |

Variant classification was performed according to ACMG/AMP guidelines. VUS findings were interpreted in the context of clinical phenotype.

Abbreviations: WES, whole exome sequencing; CES, clinical exome sequencing; TNGS, targeted next-generation sequencing; Het, heterozygous; Hom, homozygous; Pd, previously described; N, novel; VUS, variant of uncertain significance; SS, Sanger sequencing.

**Supplementary Table 2. Logistic regression analysis of serum IgM concentrations for prediction of outcomes**

| **Outcomes/Parameters** | **B** | **SE** | **OR (95% CI)** | **p** |
| --- | --- | --- | --- | --- |
| **Recurrent infections** | -0.017 | 0.041 | 0.98 (0.91–1.07) | 0.675 |
| **Autoimmune disease** | -0.006 | 0.042 | 0.99 (0.92–1.08) | 0.885 |
| **Dermatological involvement** | -0.025 | 0.041 | 0.98 (0.90–1.06) | 0.552 |
| **Endocrine disorder** | 0.036 | 0.042 | 1.04 (0.96–1.13) | 0.390 |
| **Neurological disorder** | 0.097 | 0.056 | 1.10 (0.99–1.23) | 0.085 |
| **Gastrointestinal disease** | -0.024 | 0.047 | 0.98 (0.89–1.07) | 0.614 |
| **Hepatobiliary disease** | 0.011 | 0.047 | 1.01 (0.92–1.11) | 0.810 |
| **Respiratory disease** | -0.016 | 0.041 | 0.98 (0.91–1.07) | 0.695 |
| **Lymphoproliferative disease** | -0.023 | 0.049 | 0.98 (0.89–1.08) | 0.638 |
| **Malignancy** | -0.026 | 0.057 | 0.97 (0.87–1.09) | 0.640 |
| **Osteoporosis** | -0.029 | 0.043 | 0.97 (0.89–1.06) | 0.496 |
| **Allergic disease** | -0.018 | 0.04 | 0.98 (0.91–1.06) | 0.658 |
| **Nephrological disease** | -0.008 | 0.08 | 0.99 (0.85–1.16) | 0.920 |
| **Vitamin deficiency** | 0.01 | 0.043 | 1.01 (0.93–1.10) | 0.808 |
| **Isohemagglutinin positivity** | 0.095 | 0.06 | 1.10 (0.98–1.24) | 0.111 |
| **Tetanus vaccine response** | 0.006 | 0.061 | 1.01 (0.89–1.13) | 0.920 |
| **Pneumococcal vaccine response** | -0.004 | 0.103 | 1.00 (0.81–1.22) | 0.970 |
| **Sex** | -0.026 | 0.041 | 0.97 (0.90–1.06) | 0.524 |
| **Consanguinity** | -0.21 | 0.089 | 0.81 (0.68–0.97) | **0.018** |

Abbreviations: B, regression coefficient; SE, standard error; OR, odds ratio; CI, confidence interval. Significant associations are highlighted in bold.

**Supplementary Table 3. ROC analysis of Highest IgM for clinical outcomes**

| **Clinical parameter** | **N (pos/neg)** | **AUC** | **95% CI** | **Cut-off (mg/dL)** | **Sensitivity** | **Specificity** | **Youden index** |
| --- | --- | --- | --- | --- | --- | --- | --- |
| **Lymphoproliferation** | 43 (10/33) | 0.574 | 0.353–0.763 | 27.0 | 0.7 | 0.545 | 0.245 |
| **Malignancy** | 43 (7/36) | 0.573 | 0.296–0.803 | 27.0 | 0.714 | 0.528 | 0.242 |
| **Osteoporosis** | 43 (16/27) | 0.569 | 0.386–0.744 | 28.0 | 0.688 | 0.519 | 0.206 |
| **Dermatologic Disease** | 43 (18/25) | 0.547 | 0.367–0.717 | 28.0 | 0.667 | 0.52 | 0.187 |
| **Respiratory Disease** | 43 (17/26) | 0.544 | 0.352–0.717 | 34.0 | 0.765 | 0.346 | 0.111 |
| **Frequent Infections** | 43 (25/18) | 0.534 | 0.345–0.701 | 40.0 | 0.96 | 0.222 | 0.182 |
| **Gastroenterologic Disease** | 43 (11/32) | 0.521 | 0.328–0.711 | 31.0 | 0.727 | 0.438 | 0.165 |
| **Allergic Disease** | 43 (22/21) | 0.506 | 0.331–0.685 | 35.0 | 0.818 | 0.381 | 0.199 |
| **Autoimmune** | 43 (16/27) | 0.492 | 0.323–0.665 | 28.0 | 0.625 | 0.481 | 0.106 |
| **Nephrologic Disease** | 43 (3/40) | 0.487 | 0.238–0.756 | 36.0 | 1.0 | 0.275 | 0.275 |
| **Vitamin Deficiency** | 43 (14/29) | 0.482 | 0.279–0.662 | 41.0 | 1.0 | 0.103 | 0.103 |
| **Hepatobiliary Disease** | 43 (10/33) | 0.480 | 0.264–0.683 | 28.0 | 0.6 | 0.455 | 0.055 |
| **Prophylactic Antibiotic Use** | 43 (17/26) | 0.430 | 0.248–0.609 | 28.0 | 0.588 | 0.462 | 0.05 |
| **Endocrinologic Disease** | 43 (15/28) | 0.402 | 0.235–0.571 | 35.0 | 0.8 | 0.321 | 0.121 |
| **Neurologic Disease** | 43 (7/36) | 0.286 | 0.073–0.506 | 15.0 | 0.0 | 1.0 | 0.0 |
| **Isohemagglutinin positivity** | 29 (10/19) | 0.679 | 0.466–0.892 | 33.5 | 0.70 | 0.737 | 0.437 |
| **Tetanus Vaccine Response** | 36 (30/6) | 0.447 | 0.237–0.658 | 33.5 | 0.40 | 0.833 | 0.233 |
| **Pneumococcal Vaccine Response** | 22 (20/2) | 0.525 | 0.207–0.843 | 34.5 | 0.35 | 1.00 | 0.35 |

The table summarizes AUC values with 95% confidence intervals (CI), optimal cut-off values (mg/dL), sensitivity, specificity, and Youden index for prediction of each clinical complication and treatment outcome.

Abbreviations: AUC, area under the curve; CI, confidence interval

**Supplementary Table 2. ROC analysis of Highest IgM for clinical and immunological outcomes**

| **Outcomes/Parameters** | **N (pos/neg)** | **AUC** | **95% CI** | **Cut-off (mg/dL)** | **Sensitivity** | **Specificity** | **Youden index** |
| --- | --- | --- | --- | --- | --- | --- | --- |
| **Lymphoproliferation** | 43 (10/33) | 0.574 | 0.353–0.763 | 27.0 | 0.7 | 0.545 | 0.245 |
| **Malignancy** | 43 (7/36) | 0.573 | 0.296–0.803 | 27.0 | 0.714 | 0.528 | 0.242 |
| **Osteoporosis** | 43 (16/27) | 0.569 | 0.386–0.744 | 28.0 | 0.688 | 0.519 | 0.206 |
| **Dermatologic Disease** | 43 (18/25) | 0.547 | 0.367–0.717 | 28.0 | 0.667 | 0.52 | 0.187 |
| **Respiratory Disease** | 43 (17/26) | 0.544 | 0.352–0.717 | 34.0 | 0.765 | 0.346 | 0.111 |
| **Frequent Infections** | 43 (25/18) | 0.534 | 0.345–0.701 | 40.0 | 0.96 | 0.222 | 0.182 |
| **Gastroenterologic Disease** | 43 (11/32) | 0.521 | 0.328–0.711 | 31.0 | 0.727 | 0.438 | 0.165 |
| **Allergic Disease** | 43 (22/21) | 0.506 | 0.331–0.685 | 35.0 | 0.818 | 0.381 | 0.199 |
| **Autoimmune** | 43 (16/27) | 0.492 | 0.323–0.665 | 28.0 | 0.625 | 0.481 | 0.106 |
| **Nephrologic Disease** | 43 (3/40) | 0.487 | 0.238–0.756 | 36.0 | 1.0 | 0.275 | 0.275 |
| **Vitamin Deficiency** | 43 (14/29) | 0.482 | 0.279–0.662 | 41.0 | 1.0 | 0.103 | 0.103 |
| **Hepatobiliary Disease** | 43 (10/33) | 0.48 | 0.264–0.683 | 28.0 | 0.6 | 0.455 | 0.055 |
| **Endocrinologic Disease** | 43 (15/28) | 0.402 | 0.235–0.571 | 35.0 | 0.8 | 0.321 | 0.121 |
| **Neurologic Disease** | 43 (7/36) | 0.286 | 0.073–0.506 | 15.0 | 0.0 | 1.0 | 0.0 |
| **Isohemagglutinin positivity** | 29 (10/19) | 0.679 | 0.466–0.892 | 33.5 | 0.70 | 0.737 | 0.437 |
| **Tetanus Vaccine Response** | 36 (30/6) | 0.447 | 0.237–0.658 | 33.5 | 0.40 | 0.833 | 0.233 |
| **Pneumococcal Vaccine Response** | 22 (20/2) | 0.525 | 0.207–0.843 | 34.5 | 0.35 | 1.00 | 0.35 |

The table summarizes AUC values with 95% confidence intervals (CI), optimal cut-off values (mg/dL), sensitivity, specificity, and Youden index for prediction of each clinical complication and treatment outcome.

Abbreviations: AUC, area under the curve; CI, confidence interval; IGRT, immunoglobulin replacement therapy
