# Supplementary material for: Low IgM Levels in Adult IEI: Classification Challenges and Clinical Implications
Source: J Clin Immunol. 2026 Apr 14;46(1):54. doi: 10.1007/s10875-026-02017-x (PMC13186790; doi:10.1007/s10875-026-02017-x)
Supplement: Supplementary file 2 — (DOCX 46.0 KB) [file 10875_2026_2017_MOESM2_ESM.docx]

**Supplementary Table 1. Genetic variants identified in patients with reduced serum IgM (exploratory analysis)**

| **Patient** | **Key Clinical Features** | **Method** | ***Gene*** | **Transcript** | **Zygosity** | **cDNA** | **Protein** | **Prevalence** | **ACMG** | |
| --- | --- | --- | --- | --- | --- | --- | --- | --- | --- | --- |
| P1 | Recurrent infections,  Bullous pemphigoid,  Lung carcinoma | CES | *TNFRSF13B*  *ASXL1* | *NM_012452.3*  *NM_015338.6* | Het  Presumed somatic mutation | c.204dup  c.2083C>T | p.Leu69ThrfsTer12  p.Gln695Ter | Pd  Novel | Pathogenic  Likely pathogenic (PVS1, PM2) |  |
| P2 | Recurrent infections, Chronic urticaria,  Bronchiectasis | CES | *FOXF1* | *NM_001451.3* | Het | c.1070A>T | p.His357Leu | Pd | Pathogenic |  |
| P3 | Recurrent infections,  Allergic rhinitis,  Chronic myeloid leukemia | CES | *CCDC40*  *PLCG2*  *CFTR* | *ENST00000374877*  *NM_002661.5*  *NM_000492.4* | Hom  Het  Het | c.2852_2883del  c.3793T>A  c.3454G>C | p.Gln951ArgfsTer132  p.Ser1265Thr  p.Asp1152His | Novel  Novel  Pd | Likely pathogenic (PVS1, PM2)  VUS (PM2, BP4)  Pathogenic |  |
| P4 | Lymphadenopathy,  Alport syndrome | CES | *COL4A4*  *COL4A4* | *NM_000092.5*  *NM_000092.5* | Het  Het | c.2320G>C  c.4394G>A | p.Gly774Arg  p.Gly1465Asp | Pd  Pd | Pathogenic  Pathogenic |  |
| P6 | Chronic urticaria,  Drug allergy,  Tryptase:5.99 µg/L(normal) | TNGS | *KIT* | *NM_000222.3* | Het | c.2410C>T | p.Arg804Trp | Novel | VUS (PM2, PM1) |  |
| P12 | Oral kandidiyazis | WES | *PTPN22* | *NM_015967.7* | Het | c.1411T>A | p.Tyr471Asn | Novel | VUS (PM2, BP4) |  |
| P13 | Recurrent infections,  Lymphadenopathy,  Evans syndrome,  Deep vein thrombosis | TNGS | *TREX1* | *NM_033629.6* | Het | c.-26-1G>A |  | Pd | Pathogenic |  |
| P15 | Recurrent infections | TNGS |  |  |  |  |  |  | No pathogenic variant identified |  |
| P16 | Recurrent infections,  Graves’ disease,  Lymphadenopathy,  Allergic rhinitis | CES |  |  |  |  |  |  | No pathogenic variant identified |  |
| P17 | Recurrent infections | TNGS |  |  |  |  |  |  | No pathogenic variant identified |  |
| P19 | Lymphadenopathy,  Colon cancer,  Allergic rhinitis | CES | *MAPK8IP1* | *NM_005456.4* | Het | c.1252T>C | p.Ser418Pro | Novel | VUS (PM2) |  |
| P20 | Recurrent infections,  Asthma | CES |  |  |  |  |  |  | No pathogenic variant identified |  |
| P25 | Asthma,  Allergic rhinitis,  Congenital dysfibrinogenemia | WES | *TNFRSF13B*  *FGA* | *NM_012452.3*  *NM_021871.4* | Het  Het | c.310T>C  c.167C>G | p.Cys104Arg  p.Ser56Cys | Pd  Novel | Likely pathogenic (PM2, PM5, PP3, PP5)  VUS (PM2) |  |
| P26 | Recurrent infections,  Asthma, Allergic rhinitis  Lymphadenopathy | CES | *CFTR*  *CFTR* | *NM_000492.4*  *NM_000492.4* | Het  Het | c.5A>C  c.3154T>G | p.Gln2Pro  p.Phe1052Val | Novel  Novel | VUS (PM2, PP2, PP3)  Likely pathogenic (PM1, PP2, PM2, PP3, PP5) |  |
| P27 | Recurrent infections,  Chronic diarrhea,  Drug allergy | CES |  |  |  |  |  |  | No pathogenic variant identified |  |
| P29 | Non-Hodgkin lymphoma,  Contact dermatitis | CES |  |  |  |  |  |  | No pathogenic variant identified |  |
| P30 | Recurrent infections,  Lung carcinoma,  Drug allergy,  Allergic rhinitis,  Oral ulcers | WES | *ELANE*  *MVK*  *MVK* | *NM_001972.4*  *NM_000431.4*  *NM_000431.4* | Het  Het  Het | c.781G>C  c.803T>C  c.1129G>A | p.Asp261His  p.Ile268Thr  p.Val377Ile | Novel  Pd  Pd | VUS (PM2, PP2, BP4)  Pathogenic  Pathogenic |  |
| P31 | Recurrent infections,  Asthma,  Atopic dermatitis,  Lymphadenopathy,  Horseshoe kidney | WES | *TNFRSF13B*  *MVK* | *NM_012452.3*  *NM_000431.4* | Het  Het | c.418G>A  c.928G>A | p.Glu140Lys  p.Val310Met | Pd  Pd | Pathogenic  Pathogenic |  |
| P32 | Lymphadenopathy,  Drug allergy | CES | *MEFV*  *MEFV*  *C9* | *ENST00000219596*  *ENST00000219596*  *ENST00000263408.5* | Het  Het  Het | c.2080A>G  c.442G>C  c.162C>A | p.Met694Val  p.Glu148Gln  p.Cys54Ter | Pd  Pd  Pd | Pathogenic  Pathogenic  Pathogenic |  |
| P33 | Recurrent infections,  Nephrolithiasis,  Myasthenia gravis,  Panniculitis,  Primary biliary cholangitis | CES | *FOXN1*  *ABCC8* | *NM_001369369.1*  *NM_000352.6* | Het  Het | c.1154T>C  c.1252T>C | p.Ile385Thr  p.Cys418Arg | Novel  Pd | VUS (PM2)  Likely benign (PM2, BS2, PM1, PP2,PP3, BP6) |  |
| P35 | Allergic rhinitis | SS | *CD19* | *NM_001770.6* | Het | c.648_649delTG | p.Val217AlafsTer6 | Novel | Likely pathogenic  (PVS1, PM2) |  |
| P36 | Contact dermatitis,  Pruritic plaques,  IgE ≈10,000 IU/mL, Eosinophilia | WES | *STAT6* | *NM_003153.5* | Het | DEL: chr12:57100681-57100716 |  | Novel | VUS (0.15) |  |
| P38 | Multiple sclerosis, Family history of cirrhosis | CES | *PRX*  *CASP10* | *NM_181882.3*  *NM_032977.4* | Het  Het | c.122G>A  DEL: chr2:201203439-201209817 | p.Gly41Glu | Novel  Novel | VUS (PM2)  VUS (0.15) |  |
| P39 | Recurrent infections,  Lymphadenopathy,  Prostate cancer  Allergic rhinitis | CES | *NFKB2* | *NM_001322934.2* | Het | c.1343C>T | p.Ala448Val | Novel | VUS (PM2, PP2, BP4) |  |
| P40 | Oral candidiasis,  Seborrheic dermatitis | CES | *CARD9* | *NM_052813.5* | Het | c.1434+7C>A |  | Novel | VUS (PM2, BP4) |  |
| P43 | Recurrent infections,  Lymphadenopathy,  Spongiotic dermatitis,  Stasis dermatitis,  EBV viremia | CES | *DOCK8*  *KRT6C* | *NM_203447.4*  *NM_173086.5* | Hom  Het | c.6064A>G  c.1156C>T | p.Met2022Val  p.Arg386Cys | Novel  Novel | VUS (PM2, BP6)  VUS (PM2, PP3) |  |

Variant classification was performed according to ACMG/AMP guidelines. VUS findings were interpreted in the context of clinical phenotype.

Abbreviations: WES, whole exome sequencing; CES, clinical exome sequencing; TNGS, targeted next-generation sequencing; Het, heterozygous; Hom, homozygous; Pd, previously described; VUS, variant of uncertain significance; SS, Sanger sequencing.

**Supplementary Table 2. Exploratory logistic regression analysis evaluating associations between serum IgM concentrations and selected clinical and laboratory parameters**

| **Outcomes/Parameters** | **B** | **SE** | **OR (95% CI)** | **p** |
| --- | --- | --- | --- | --- |
| **Recurrent Infections** | -0.017 | 0.041 | 0.98 (0.91–1.07) | 0.675 |
| **Autoimmune Disease** | -0.006 | 0.042 | 0.99 (0.92–1.08) | 0.885 |
| **Dermatological Involvement** | -0.025 | 0.041 | 0.98 (0.90–1.06) | 0.552 |
| **Endocrine Disorder** | 0.036 | 0.042 | 1.04 (0.96–1.13) | 0.390 |
| **Neurological Disorder** | 0.097 | 0.056 | 1.10 (0.99–1.23) | 0.085 |
| **Gastrointestinal Disease** | -0.024 | 0.047 | 0.98 (0.89–1.07) | 0.614 |
| **Hepatobiliary Disease** | 0.011 | 0.047 | 1.01 (0.92–1.11) | 0.810 |
| **Respiratory Disease** | -0.016 | 0.041 | 0.98 (0.91–1.07) | 0.695 |
| **Lymphoproliferative Disease** | -0.023 | 0.049 | 0.98 (0.89–1.08) | 0.638 |
| **Malignancy** | -0.026 | 0.057 | 0.97 (0.87–1.09) | 0.640 |
| **Osteoporosis** | -0.029 | 0.043 | 0.97 (0.89–1.06) | 0.496 |
| **Allergic Disease** | -0.018 | 0.040 | 0.98 (0.91–1.06) | 0.658 |
| **Nephrological Disease** | -0.008 | 0.080 | 0.99 (0.85–1.16) | 0.920 |
| **Vitamin Deficiency** | 0.01 | 0.043 | 1.01 (0.93–1.10) | 0.808 |
| **Isohemagglutinin Positivity** | 0.095 | 0.060 | 1.10 (0.98–1.24) | 0.111 |
| **Tetanus Vaccine Response** | 0.006 | 0.061 | 1.01 (0.89–1.13) | 0.920 |
| **Pneumococcal Vaccine Response** | -0.004 | 0.103 | 1.00 (0.81–1.22) | 0.970 |
| **Sex** | -0.026 | 0.041 | 0.97 (0.90–1.06) | 0.524 |
| **Consanguinity** | -0.21 | 0.089 | 0.81 (0.68–0.97) | **0.018** |

Abbreviations: B, regression coefficient; SE, standard error; OR, odds ratio; CI, confidence interval. Given the limited sample size, these analyses should be interpreted as exploratory. Significant associations are highlighted in bold.

**Supplementary Table 3. Exploratory ROC analysis evaluating the discriminatory performance of serum IgM levels for selected outcomes**

| **Clinical Parameter** | **N (pos/neg)** | **AUC** | **95% CI** |
| --- | --- | --- | --- |
| **Lymphoproliferation** | 43 (10/33) | 0.574 | 0.353–0.763 |
| **Malignancy** | 43 (7/36) | 0.573 | 0.296–0.803 |
| **Osteoporosis** | 43 (16/27) | 0.569 | 0.386–0.744 |
| **Dermatologic Disease** | 43 (18/25) | 0.547 | 0.367–0.717 |
| **Respiratory Disease** | 43 (17/26) | 0.544 | 0.352–0.717 |
| **Recurrent Infections** | 43 (25/18) | 0.534 | 0.345–0.701 |
| **Gastrointestinal Disease** | 43 (11/32) | 0.521 | 0.328–0.711 |
| **Allergic Disease** | 43 (22/21) | 0.506 | 0.331–0.685 |
| **Autoimmune Disease** | 43 (16/27) | 0.492 | 0.323–0.665 |
| **Nephrological disease** | 43 (3/40) | 0.487 | 0.238–0.756 |
| **Vitamin Deficiency** | 43 (14/29) | 0.482 | 0.279–0.662 |
| **Hepatobiliary Disease** | 43 (10/33) | 0.480 | 0.264–0.683 |
| **Prophylactic Antibiotic Use** | 43 (17/26) | 0.430 | 0.248–0.609 |
| **Endocrine disease** | 43 (15/28) | 0.402 | 0.235–0.571 |
| **Neurological disease** | 43 (7/36) | 0.286 | 0.073–0.506 |
| **Isohemagglutinin Positivity** | 29 (10/19) | 0.679 | 0.466–0.892 |
| **Tetanus Vaccine Response** | 36 (30/6) | 0.447 | 0.237–0.658 |
| **Pneumococcal Vaccine Response** | 22 (20/2) | 0.525 | 0.207–0.843 |

The table summarizes area under the curve (AUC) values with 95% confidence intervals (CI) derived from exploratory ROC analyses evaluating the discriminatory performance of serum IgM levels for selected clinical and immunological outcomes. Given the limited sample size and wide confidence intervals, these analyses should be interpreted as exploratory rather than definitive assessments of predictive performance.
